# Positive Narrative Group Psychotherapy: the use of traditional fairy tales to enhance psychological well-being and growth

**DOI:** 10.1186/s13612-013-0013-0

**Published:** 2014-04-05

**Authors:** Chiara Ruini, Licia Masoni, Fedra Ottolini, Silvia Ferrari

**Affiliations:** Department of Psychology, University of Bologna, Viale Berti Pichat 5, 40127 Bologna, Italy; Department of Education Sciences, University of Bologna, Bologna, Italy; Department of Diagnostic-Clinical Medicine and Public Health, Section of Psychiatry, University of Modena and Reggio Emilia, Modena, Italy

**Keywords:** Narrative, Psychotherapy, Personal growth, Psychological well-being, Traditional fairy tales

## Abstract

**Background:**

Oral narrative strategies have rarely been applied in the positive psychology domain. Traditional folk and fairy tales are concerned with several concepts that are now scientifically investigated by research on positive psychology, such as resilience, self-realization, personal growth and meaning in life. The aim of this pilot study was to apply a new narrative approach based on fairy tales (Märchen, tales of magic, rise tales) told, discussed, and written in a group context for the purpose of promoting psychological well-being and growth.

**Methods:**

A group intervention consisting of 7 sessions was delivered to 21 women who reported adjustment disorder. The group was conducted by a folklorist and a clinical psychologist. Each session consisted of an introduction to a number of themes recurring in fairy tales as well as references to the social uses of narratives, followed by a discussion with the participants. In two concluding workshops, participants were asked to work as a group to write their own original fairy tale. Assessment pre- and post-intervention was performed with the Ryff Psychological Well-being Scale, the Kellner Symptom Questionnaire and 2 subscales of the Posttraumatic Growth Inventory *(Appreciation of Life and personal strengths).*

**Results:**

Participants reported increased personal growth, self-acceptance, and an enhanced sense of appreciation of life and personal strengths, together with decreased levels of anxiety.

**Conclusions:**

This pilot investigation suggests the feasibility and positive effect of a group intervention based on narrative strategies for promoting well-being and growth in stressed women. Considering its promising results, clinical implications and possible further applications are discussed.

## Background

The use of narrative strategies in clinical psychology and psychotherapy has a long tradition (Holmes 2000). Following Jung (1964) and his theories on archetypes and myths, Jungian psychoanalysts have applied his concepts in the treatment of various emotional disorders (Kast 1996). In the wake of this tradition, many contemporary authors underline the clinical value of employing myths and metaphors in the process of psychotherapy (Dieckmann 1997). Some clinicians have suggested that the process of psychotherapy itself could be conceptualized as a narration, within the clinical relationship between patient and therapist (White & Epston 1990; Grafanaki & McLeod 1999), both of whom listen, tell and share different stories that have meaning and clinical value in the therapeutic alliance. In this framework, patients have recently been asked to describe their psychotherapy in a narrative way, commenting on initial phases, central themes, and final sessions (Adler & McAdams 2007).

The importance of using narrative strategies in clinical domains has likewise been documented by many authors within the “narrative medicine” framework (Kleinman 1988; Riessman 1990; Pennebaker 2000; Charon 2006a). This method calls for a holistic approach to patients that encompasses not just their illness, but also their biography, personal narration and cognitive interpretation of their illness (Charon 2006b).

Similarly, White and Epston (1990) observed that individual narratives associated with psychological distress were characterized by thin and impoverished stories. They developed a narrative therapy (1990) with the aim of deconstructing dominant problem stories, incorporating previously neglected stories into the overall life story of the person, and achieving final enriched life stories (White & Epston 1990). Clinicians have developed specific methods for analyzing and interpreting their patients’ dialogues and narrations (Angus 2012; Adler 2012). Such methods have often been used to evaluate treatment outcomes (Goncalves et al. 2011; Vromans & Schweitzer 2011). This narrative approach has been widely used and proved to be effective in treating mood and anxiety disorders. (Vromans & Schweitzer 2011).

In line with this approach, the life-review intervention, mainly used with elderly populations (Broadhead 2012), may also be considered a specific application of narrative strategies in psychological interventions. In this case, individuals are asked to use journals where they record their life, describing its most salient events (Broadhead 2012; Pennebaker 2000). These techniques have recently been used for treating patients with dementia or other degenerative illnesses. Subramaniam & Woods (2012) found that individual reminiscence which involved life review containing personalised specific memory triggers resulted in some benefits for depression, well-being and social engagement among older patients with dementia. On the contrary, general reminiscence materials yielded no such benefits. Therefore, the use of specific cognitive stimuli connected with subjective emotional content appears to be particularly indicated for ageing individuals with cognitive impairments (Subramaniam & Woods 2012).

Alternatively, narrative strategies have been used with children and adolescents (Lubetsky 1989; Bennett 2008; Burns 2005). In this case, specific stories were used to help children overcome particular instances of fear, abuse, trauma, or other negative events (such as death of a family member, diagnosis of severe or chronic illnesses, etc.). In most cases, the clinician has become a writer and storyteller himself/herself, creating a set of specific stories to be used within specific clinical situations (Brandt 1983).

Finally, a widespread use of narration in the psychological sphere comes from Bettelheim’s (1976; 1987) analytic interpretations of fairy tales. Some clinicians in the psychoanalytic tradition employ folktales and their characters (hero/heroine) during sessions, as a means of discussing the tales’ symbolic values with their patients (Jacobs 2011).

Unlike Bettelheim, who refers mostly to printed tales, we chose to concentrate on archival transcripts of fairy tales collected from the oral tradition (in societies that relied on narratives as their main means of discussing reality), rather than literary or purpose-written ones (we did not use Grimm’s texts or those of other well known anthologies). Importantly, we decided to present the group with two or more orally collected versions of a selected number of traditional fairy tales, in order to explore how the attitude to universal fairy tale themes (such as separation, violence, generational conflicts, gender roles) could vary from culture to culture and family to family, reflecting the needs of those who had adapted tales for their personal use and growth. In traditional societies, fairy tales played an important psycho-educational role, providing *ad hoc* frameworks to interpret reality, to deal with personal issues and stressful events, to encourage or warn in facing life’s adversities, and to help enter adulthood: fairy tales were aimed at young adults more than children, and storytellers were mostly older and well respected members of the community who communicated their wisdom and life experience through the stories in their repertoires. The content of fairy tales deals with several concepts that are now scientifically investigated by clinical psychology and psychotherapy research, such as resilience, hope and passion (Weis & Speridakos 2011; Vallerand 2012), personal growth and meaning in life (Ruini & Fava 2012; Seligman 2002).

The aim of this work was the introduction of a specific application of narrative therapy in the psychological/psychotherapeutic domain, that makes use of the narrative structure of fairy tales, as well as the metaphors they contain, to help people solve problems in their daily life, overcome stressful events and increase their well-being. We present a pilot study with emotionally distressed women, and describe a narrative intervention based on fairy tales told and discussed in a group format.

## Methods

### Participants

The sample was recruited by advertisement by an Italian non-profit organization offering psychological support to women experiencing various kinds of stress and difficulties in life. The inclusion criteria were the female gender and a diagnosis of adjustment disorder following various stressful life events (prolonged unemployment, or work-related stress and burn-out, separation or divorce, etc.). We used a gender inclusion criterion to obtain a gender-homogeneous group, in order to facilitate the sharing and reciprocal group mirroring of life experiences.

Respondents were evaluated by a clinical psychologist using the SCID (First et al. 2002). Women presenting psychotic disorders, schizophrenia, or bipolar disorders were excluded. Of 30 women approached, 9 refused to participate or did not meet inclusion criteria. The final sample was composed of 21 women (mean age = 39.33; SD = 11.51) reporting a diagnosis of adjustment disorder. Participants signed an informed consent form prior to entering the study. The non-profit organization’s ethical committee approved the study.

## Materials

1. Symptom Questionnaire (SQ) (Kellner 1987) is a 92-item self-rating scale that yields 4 scales of distress (anxiety, depression, somatisation and hostility-irritability) and 4 scales of well-being (relaxation, contentment, physical well-being and friendliness). Each symptom scale’s score may range from 0 to 17; each well-being scale scores from 0 to 6. The SQ was previously validated in an Italian population and proved to be a sensitive instrument for detecting changes in clinical trials (Fava et al. 1983). The conventional split-half reliability of the scales in various studies was as follows: anxiety, .75 to .95 (median, .83); depression, .74 to .93 (median, .91); somatisation, .57 to .84 (median, .78); hostility, .78 to .95 (median, .89) (Kellner 1987).
2. The Psychological Well-Being Scale (PWB) (Ryff 1989) is an 84 item self-rating inventory that covers the 6 areas of psychological well-being: autonomy, environmental mastery, personal growth, positive relations with others, purpose in life and self-acceptance. Subjects respond with a 6-point Likert scale ranging from “strongly disagree” to ”strongly agree“. Responses to negatively scored items are reversed in the final scoring procedure. The PWB has already been validated in an Italian population (Ruini et al. 2003). The PWB scales also have satisfactory test-retest reliability and are inversely related to measures of psychological distress (Kellner 1987).
3. Post-traumatic Growth Inventory (PTGI) (Tedeschi & Calhoun 1996) assesses positive changes experienced after life crises. We selected two particular subscales, namely Personal Strength (4 items) and Appreciation of Life (3 items), as they contain items characterised by an existential content that fit the purpose of our study. Items are rated on a 6-point Likert scale, ranging from 0 (”did not experience this change as a result of my crisis”) to 5 (“experienced this change to a very great degree as a result of my crisis”).

### Procedures

Women were assessed pre- and post-intervention with the above self-report instruments.

### Intervention

The group intervention consisted of seven two-hour sessions, held once a week. The group was conducted by a folklorist and by a clinical psychologist. In each of the first 5 sessions (‘lectures’) a different fairy tale (or part of it), with related topics, was told and discussed with the participants. Specific topics were selected: age and gender-related conflicts and tensions among characters, the ability to cope with adversities and stressful situations, the use of inner resources and personal strengths, couple and family dynamics, the importance of cooperation (asking for and accepting help), and the role of helpers and magical gifts. The 5 lectures were articulated as follows:

1. «The heroine in traditional fairy tales»: female role models.
2. «The charming prince in traditional fairy tales»: male role models.
3. «Cinderella’s revenge»: how to discover and use inner resources inside every woman.
4. «Things Walt Disney never told us» (named after Stones (1975) article): less popular fairy tales and their symbolic meaning.
5. «Happily ever after? »: the couple in fairy tales.

During each session, after the storytelling and related explanations by the folklorist, the clinical psychologist highlighted the emotional-psychological contents of the specific fairy tales, without using psychoanalytic or Jungian approaches (Kast 1996; Dieckmann 1997). Rather, within a cognitive-behavioral framework, similarities with nowadays problems, specific coping strategies adopted by the hero/heroine of the story, and his/her emotional maturation were underlined and discussed by the group.

In the 2 final sessions (‘workshops’), participants were asked to write as a group their original fairy tale. The folklorist gave them a basic structure, based on the concept of ‘functions’ developed in fairy tale structural analysis (Propp 1968; Dundes 1982; Holbek 1987), consisting of 6 main phases: initial stressful event, test, tasks, help, fight, victory, final reward. Following this structure, at the beginning of the story the hero/heroine faces a stressful event that forces her/him to begin a physical or metaphorical journey; during such journey, he/she will be tested, thus revealing his/her nature, and rewarded with gifts; the gifts will aid him/her perform a series of difficult tasks that will lead to the elimination/solution of the initial problem, and to the achievement of a new positive or balanced phase in the hero/heroine’s life.

The participants were asked to make up and write together two tales (one for each of the two workshops), one with a female protagonist and one with a male protagonist. Given that traditional tales describe the characters through their actions and not by means of psychological descriptions, the participants were told to work together as a group at establishing a set of values that would guide the protagonist’s actions, thus defining his/her nature.

To conclude the intervention, a more informal meeting was held, in order to collect the participants’ feedbacks about the experience, and present them with printed copies of their original fairy tales.

### Statistical analysis

A general linear model (GLM) for repeated measure, with intervention as within subject factor was used to compare initial and post-treatment scores for all scales. Significance level was set at .05, two tailed.

Effect sizes were calculated using partial eta squares. Statistical Package for the Social Sciences (SPSS) Windows Software Version 19.0 was used.

Qualitative data (participants’ feedback) were also collected, through a specific focus group held during the final informal meeting at the end of the intervention. Participants were asked to report the major benefits perceived as a result of the intervention, as well as its main negative aspects. Participants’ answers were recorded and analyzed using an open coding procedure (Strauss and Corbin 1990).

## Results

Multivariate test showed a significant effect of intervention (F(12, 9) =8.406, p = .002, partial Eta squared = .918). At the end of the intervention participants reported an increased personal growth and self-acceptance (PWB), together with decreased levels of anxiety (SQ). Similarly, the two PTGI subscales of Appreciation of Life and Personal Strengths significantly increased after intervention, with large effect size values. Table 1 displays the results at univariate tests.

**Table 1:** **Modifications of distress and well-being following Positive Narrative Group Psychotherapy**

	**Pre (n = 21)**	**Post (n = 21)**	**F**	**η** ^**2**^
**PWB**				
Autonomy	61.90 ( SD = 8.46)	63.38 (SD = 8.76)	1.59	0.74
Environmental mastery	57.52 (SD = 8.07)	59.38 (SD = 9.99)	1.62	.050
Personal growth**	60.67 (SD = 10.20)	64.00 (SD = 9.69)	10.83**	.351
Positive Relations w/others	61.52 (SD = 12.734)	61.81 (SD = 11.098)	.030	.001
Purpose in life	60.00 (SD = 13.248)	60.38 (SD = 11.745)	.051	.003
Self-acceptance*	56.38 (SD = 9.615)	59.67 (SD = 9.77)	4.551*	.185
**SQ**				
Anxiety*	5.05 (SD = 3.457)	3.67 (SD = 2.497)	4.741	.192
Depression	3.48 (SD = 2.089)	2.81 (SD = 1.914)	1.931	.088
Somatic Symptoms	5.19 (SD = 3.642)	4.48 (SD = 2.994)	.622	.030
Hostility-irritability	4.33 (SD = 3.440)	4.10 (SD = 4.403)	.048	.002
**PTG**				
Personal strength**	14.00 (SD = 3.286)	15.95 (SD = 2.801)	29.133**	. 593
Appreciation of life**	9.38 (SD = 4.45)	10.62 (SD = 4.08)	36.150**	.644

*Note.* *p ≤ 0.05, **p ≤ 0.01 significativity level at univariate model, SD = Standard deviation, PWB = Psychological Well-being Scales, SQ = Symptom Questionnaire, PSI = Psychosocial Index , PTG = Posttraumatic Growth Inventory.

### Feedback from participants

In the final session, participants revealed that the main benefits from intervention were: improved awareness of personal strengths; improved acceptance of difficulties and stressors as part of human life; increased goals in life; new meaning in daily life; new friends, sharing common experiences and stressors; increased awareness of common cultural background. A few negative aspects were also reported, related to excessive shortness of the intervention, location, and meetings timetable.

## Discussion

In this pilot study, we tested a new narrative approach, based on traditional fairy tales, with the aim of helping distressed women overcome their difficulties and increase their psychological well-being. The preliminary results suggest that at the end of the group intervention participants reported decreased levels of anxiety and improvements in personal growth, self-acceptance, appreciation of life and personal strengths.

Therefore, this new narrative approach seems to yield positive effects, both on symptoms and on well-being. These positive effects are confirmed by large effect size, particularly on anxiety symptomatology and existential dimensions (appreciation of life and personal strengths). These could be considered promising results, in view of the novelties introduced by this approach.

Even though previous authors have underlined the importance of fairy tales and storytelling for promoting the healing process of individuals (Burns 2001; 2005, 2007; Dieckmann 1986; 1997; Mills et al. 2001; Pearson et al. 2013, Warner 2006), in our intervention fairy tales were not used for their symbolic meaning, or for psychoanalytic purposes. Rather, the novelty of this narrative approach is based on addressing the psycho-educational content of traditional fairy tales, their problem-solving approach and the process of maturation experienced by their characters. Participants were given the means to discuss and interpret stories, traditional ones and their own, by themselves. These issues could be subsumed under the rubric of resilience capacities, meaning, wisdom and character strengths, which are psychological topics that have been received increasing attention in recent years (Proyer & Ruch 2011). Several positive psychology interventions, also with clinical populations, have been validated and have proven to be effective in increasing life satisfaction, positive emotions, resilience and well-being (Seligman 2002; Ruini & Fava 2012; Cope 2010). Therefore, positive group narrative psychotherapy is in line with this trend of research.

Another significant novelty of our group narrative intervention concerns the fact that fairy tales were not simply read and discussed with clients, or interpreted for their symbolic meaning (for instance, asking clients to report their favorite fairy tales or characters), which is the case for many other narrative approaches. Rather, the narrative structure of the fairy tale (Propp 1968) was used to help clients write their own story. This very active part of the intervention aimed at stimulating playfulness, creativity, exercising in finding new logical and causal connections between events, and increasing group cohesion (Proyer & Ruch 2011; Treadwell et al. 2011); at the same time, it contributed to improved awareness of problems in life and more flexible problem-solving techniques. Creativity, self-awareness, and flexibility (Kashdan & Rottenberg 2010) are the focus of the majority of positive interventions nowadays (Seligman 2002). In particular, based on direct observation of participants during the workshops and subsequent feedback and comments, we think that this active creation of the story could be considered the most effective ingredient of the described new narrative approach. Indeed, in the process of writing their original fairy tale, participants could work at creating metaphors, rather than just interpreting them. The working approach was: “I would like to convey this characteristic or feeling, so what action/symbol could I employ in order to do so?”, rather than “This event has occurred in the story: what could it mean, what could it stand for?”. As a result, they were able to develop their own symbolic language. Furthermore, their emotions were projected in a fictional, impossible setting, and this provided them with the right distance from problems, connected with a more effective cognitive engagement in problem solving. This emotional distance is an active ingredient also of Wisdom-Psychotherapy, a recently validated form of group psychotherapy for addressing embitterment disorders (Linden et al. 2011).

## Conclusions

In conclusion, in this preliminary study we used the fairy tales’ important educational role and psycho-social implications to help distressed women address their emotional distress and increase their personal growth. According to our study, fairy tales are suitable for use in a group setting (Treadwell et al. 2011) and can easily support the work of psychotherapy, particularly the cognitive-behavioral format (Blenkiron 2005).

This study is limited by its small, self-selected sample, its preliminary nature and naturalistic design, the lack of a controlled condition, and the use of self-report assessments only.

However, this pilot investigation suggests the feasibility and positive effect of a group intervention based on narrative strategies for promoting well-being and growth in stressed women. Considering its promising results, further controlled studies, with larger samples and in clinical settings, are recommended.
